# Optimal Behavior is Easier to Learn than the Truth

**DOI:** 10.1007/s11023-016-9389-y

**Published:** 2016-02-03

**Authors:** Ronald Ortner

**Affiliations:** Department Mathematik und Informationstechnologie, Montanuniversität Leoben, Franz-Josef-Straße 18, 8700 Leoben, Austria

**Keywords:** Markov decision processes, Truth, Reinforcement learning, Regret

## Abstract

We consider a reinforcement learning setting where the learner is given a set of possible models containing the true model. While there are algorithms that are able to successfully learn optimal behavior in this setting, they do so without trying to identify the underlying true model. Indeed, we show that there are cases in which the attempt to find the true model is doomed to failure.

## Introduction

In reinforcement learning problems, an agent acts in an unknown environment that allows the agent to take actions that are followed by a response of the environment. The paradigm for representing such reinforcement learning problems are *Markov decision processes*, where starting in some initial state $s_1$, the agent at time steps $t=1,2,\ldots$ chooses an action $a_t$ from a set of actions *A*, obtains a random reward depending on the current state $s_t$ and the chosen action $a_t$, and then moves to state $s_{t+1}$ according to transition probabilities that also depend on the state-action pair $(s_t,a_t)$. Formally, a Markov decision process is defined as follows.

### **Definition 1**

A *Markov decision process* (MDP) ${\mathcal {M}}$ consists of a set of *statesS* with some distinguished initial state $s_1$, a set of *actionsA*, reward distributions with mean *r*(*s*, *a*) for the reward when choosing action *a* in state *s*, and transition probabilities $p(s'|s,a)$ for the probability of moving to state $s'$ when choosing action *a* in state *s*.

However, in many practical reinforcement learning problems (e.g. applications in robotics) the underlying state space can either be huge or even unknown. Thus, a chess playing robot may be confronted with the same board position on two different occasions, but the respective video signals of the board may be different. Thus, it makes sense to distinguish between *observations* and *states*. In more complex scenarios as we will consider here, the agent only has direct access to observations (like the video signal of the robot) but has no information about the underlying state (the respective board position). In the motivating example, states could be considered as sets of observations (corresponding to the same state), or equivalently, mappings from the set of observations *O* to a state space *S*. Without prior knowledge the agent has to consider various such *models* that map observations to states. In our example, the *true* model would map all images showing the same board position to the same state, but of course when learning from scratch it is not clear that this is the correct model. Rather, it seems that the learner has to learn the true model or a good approximation of it as well. Actually, we will consider more general models that need not aggregate observations but more generally *histories*, that is, the sequences of observations, rewards, and chosen actions. This notion of models has been introduced by Hutter (2009).

### **Definition 1**

In a reinforcement learning problem, the *history*$h_t$ after *t* time steps is the sequence $h_t:=o_1,a_1,r_1,o_2,a_2,r_2,\ldots , a_t,r_{t},o_{t+1}$ of observations $o_{\tau } \in O$, collected rewards $r_{\tau } \in {\mathbb {R}}$, and chosen actions $a_{\tau }\in A$ at time steps $\tau =1,\ldots ,t$.

### **Definition 2**

A *model*$\phi :H\rightarrow S_\phi$ is a mapping from the set of histories *H* to the state space $S_\phi$ (under $\phi$) and maps each history $h\in H$ to a state $\phi (h)$ in the state space $S_\phi$.

That is, similar to our motivating example, a model assigns a respective state to each situation in which the agent can find himself in (i.e., a history). In the example of the chess playing robot, the observation of the board is actually not always sufficient to decide whose move it is, and one has to take into account also the recent history of observations to determine the correct state of the game. Note that the notion of *model* in Definition 2 is still rather modest, as we do not demand that a model also specifies the precise values of the mean rewards and transition probabilities of all state-action pairs, which would make things obviously much harder.

Now, we assume that there is a *true* model $\varphi _\mathrm{true}$ that maps histories to states with respect to which the environment behaves like a Markov decision process.^1^ However, this model is unknown to the learner. Rather, the learner has a set of possible models $\Phi$, each mapping histories to states, at her disposal which we assume to contain the true model $\varphi _\mathrm{true}$.

In this paper we are interested in the following questions: Is it possible to identify the true model? Can an agent learn to behave optimally in the underlying true Markov decision process? Is the identification of the true model a necessary prerequisite for optimal behavior? Surprisingly, it turns out that not only is the answer to the latter question negative, in general it is not possible to identify the true model, while it is still possible to learn optimal behavior.

## Learning Optimal Behavior is Possible (and Not Much More Difficult than in the MDP Setting)

### MDP Preliminaries

We are interested in learning optimal behavior in the setting introduced in the previous section, competing with an optimal strategy that maximizes the collected rewards. Before making this more precise, we will introduce some assumptions concerning the formal framework. First, in the following we assume that the random rewards are bounded. Intuitively, if we allowed rewards to be unbounded, any learning algorithm may miss a very large reward at a particular time step, a loss which it may not be able to recover anymore. For the sake of simplicity we assume the rewards to be bounded in the unit interval [0, 1], which can be easily achieved by rescaling the rewards in a suitable way.

Further, while there are a few theoretical results for reinforcement learning in MDPs with infinite state space under some additional assumptions (see e.g. Ortner and Ryabko 2012; Lakshmanan et al. 2015), we also assume that the state space and the action space are finite. Note however, that the set of observations is allowed to be infinite, and it may well be that the learner sees no observation twice.

We continue with some preliminary theory on Markov decision processes, see e.g. (Puterman 1994). A *(stationary) policy* on an MDP ${\mathcal {M}}$ fixes for each state a respective action, that is, it is a mapping $\pi : S \rightarrow A$. The *average reward* of such a policy $\pi$ is defined as

$$\begin{aligned} \rho ({\mathcal {M}},\pi ) \;:=\; \lim _{T\rightarrow \infty } \frac{1}{T} \sum _{t=1}^{T} {\mathbb {E}}\left [r\big (s_t,\pi (s_t)\big )\right ], \end{aligned}$$

where $s_t$ denotes the random state that is visited at step *t* when choosing actions according to policy $\pi$. An *optimal policy*$\pi ^*$ on ${\mathcal {M}}$ maximizes the average reward, that is, $\rho ({\mathcal {M}},\pi ^*)\ge \rho ({\mathcal {M}},\pi )$ for all policies $\pi$. It can be shown that the average reward cannot be increased by using *non-stationary* policies (that may choose different actions when visiting the same state on different time steps), so that the restriction to stationary policies in the definition of $\pi ^*$ is justified. However, a learning algorithm may still perform optimally employing a nonstationary policy. Now, by learning optimal behavior in an MDP, we mean that an agent operating on an MDP succeeds in converging to an optimal (possibly nonstationary) policy that maximizes the average reward.

### Learning Optimal Behavior in MDPs

There are several algorithms such as E$^3$ (Kearns and Singh 2002), R-max (Brafman and Tennenholtz 2002), or UCRL (Jaksch et al. 2010) that are able to learn optimal behavior in an MDP setting when the learner only has knowledge of the state and action space of the underlying MDP. The E$^3$ algorithm (E$^3$ stands for “explicit explore or exploit”) maintains a partial model^2^ of the underlying MDP and plays an exploration strategy in states that are not sufficiently known, while exploiting (i.e., playing the optimal policy in the estimated model) in known states. R-max and UCRL are based on the idea of “optimism in the face of uncertainty”. Thus, R-max maintains a model in which all states that have not been visited sufficiently often have maximal possible reward (hence the name “R-max”) and plays the optimal policy in this model. The UCRL algorithm refines this idea by using confidence intervals for rewards and transition probabilities and assuming the most optimistic parameters^3^ in these confidence intervals. Again, the algorithm then chooses the optimal policy based on this optimistic model of the MDP.^4^

The theoretical results for these algorithms go beyond simple convergence to an optimal policy for time $T\rightarrow \infty$. Indeed, for all the mentioned algorithms there are also guarantees on their finite-time behavior. Thus, the *sample-complexity bounds* for E$^3$ (Kearns and Singh 2002) and R-max (Brafman and Tennenholtz 2002) give bounds on the number of time steps after which the algorithm is *probably approximately correct (PAC)*, that is, close to optimal with high probability.^5^ For UCRL there are even bounds on the *regret* the algorithm suffers with respect to an optimal policy after any *T* steps. More precisely, the *regret* of an algorithm is defined as follows.

#### **Definition 3**

The regret of an algorithm in an MDP ${\mathcal {M}}$ after *T* steps is defined as the difference between the total reward of an optimal policy and the accumulated reward of the algorithm, that is,

$$\begin{aligned} T \cdot \rho ({\mathcal {M}},\pi ^*)-\sum _{t=1}^T r_t, \end{aligned}$$

where $r_t$ is the (random) reward obtained by the algorithm at step *t*.

For the UCRL algorithm it was shown (Jaksch et al. 2010) that the regret after any *T* steps is upper bounded by a term of order $\sqrt{T}$ (ignoring parameters of the MDP that also appear in the bound^6^), that is, the per-step regret of UCRL converges to 0 at a rate of $\frac{1}{\sqrt{T}}$.

### Reinforcement Learning with Model Selection

The question of learning optimal behavior in the model selection setting introduced in Sect. 1 was first considered by Hutter (2009). A bit later, Maillard et al. (2012) gave the first regret bounds in this setting. Using the UCRL algorithm as a subroutine, the suggested BLB algorithm computes confidence intervals for the regret of each model, and chooses the model for which the lower value of the confidence interval is maximal (accordingly, BLB stands for “best lower bound”). The regret of BLB after any *T* steps was shown to be upper bounded by a term of order $\root 3 \of {T^2}$ (again disregarding other parameters than *T*). Although learning in the model selection setting looks more difficult, as the learner seems to have the additional task of identifying the right model, recently an algorithm was presented by Maillard et al. (2013) which has the same regret rate of $\sqrt{T}$ as UCRL in the MDP setting.^7^ This algorithm (called OMS for “optimistic model selection”) actually does not try to identify the true model, but chooses the model on an optimistic basis (just like the UCRL algorithm in the MDP case). That is, OMS chooses the model which promises the highest reward (based on the estimates so far). Even if a non-Markov model is chosen, the algorithm compares the collected rewards to a respective (fictitious) Markov model. The model is rejected if it performs below the threshold determined by this Markov model. In particular, this means that the algorithm is willing to act according to a wrong non-Markov model as long as the rewards collected under this model are high enough, that is, are indistinguishable from the rewards a Markov model would give. Thus, the truth of the applied model is not important for the algorithm.

## Finding the True Model May be Impossible

The question of identifying the true model in a similar setting as considered here has already been investigated by Hallak et al. (2013).^8^ It is shown that under certain assumptions the true model indeed can be identified in the long run. However, the assumptions made by Hallak et al. (2013) are so strong that they will be satisfied only in a small fraction of learning problems and rarely hold in applications. Thus, on one hand it is assumed that all state-action pairs are visited infinitely often when time $T\rightarrow \infty$, on the other hand, the policy applied shall be constant. Thus, the existence of an *ergodic* policy is assumed. Such ergodic policies however do not even exist in simple scenarios such as when all transitions are deterministic. On the other hand, even if ergodic policies exist, without knowing the correct model in advance it is not clear that such a policy can be identified. Moreover, it is assumed that each observation has only one possible predecessor state, and that the models given to the learner are *hierarchical*, that is, the models $\phi _1,\ldots ,\phi _n$ given to the learner are such that each $\phi _i$ is a refinement^9^ of all models $\phi _j$ with $j<i$. Obviously, the latter restriction on the model structure is particularly strong.

The following example, violating the condition of hierarchical models, shows that identification of the true model is not always possible.

### *Example 1*

Assume a model selection problem where the learner is given four models $\phi _1$, $\phi _2$, $\phi _3$, $\phi _4$. The action set contains two actions $a_1$, $a_2$, and the first observation made by the learner is $o_1$. Assume that $\phi _1$ and $\phi _2$ coincide on all histories (that is, map all histories to the same state) except the history $o_1a_2$, which is mapped to different states under $\phi _1$ and $\phi _2$. Also assume that $\phi _3$ and $\phi _4$ coincide on all histories except the history $o_1a_1$.

Then, if the learner chooses $a_1$ as her first action, obviously it is not possible to decide between the models $\phi _1$ and $\phi _2$, as the only history where the two models differ never appears. Similarly, if the learner first chooses $a_2$, she cannot decide between the models $\phi _3$ and $\phi _4$. Consequently, independent of which action the learner chooses there are two indistinguishable models. Since each of the models can be made to be the true model, the example shows that there are learning problems in which it is impossible to identify the true model.

While Example 1 gives an extreme case, it is easy to weaken it. Indeed, whenever two models differ from each other on sufficiently few histories, no statistical test will be able to distinguish them, even if—unlike in Example 1—the respective histories are observed by the learner.

Even in more convenient settings where models differ more strongly, the identification of the true model constitutes a difficult problem: The learner has to determine whether samples collected in the same state (according to some model) and given an action are really generated by the same process. Similar problems have been e.g. considered by Navarro et al. (2004) or Wagenmakers et al. (2004), cf. also the discussion in Sect. 4.2 below.

## Discussion

### Other Notions of Learning

It is important to note the difference between the reinforcement learning setting we consider and other learning scenarios that appear in the machine learning literature. Historically, the first formal setting for learning—introduced by Gold (1967)—was that of learning recursive functions from their graphs. In this setting, different notions of learning in the limit have been considered (cf. e.g. Case and Smith (1983)) that imply different classes of recursive functions that turn out to be learnable. An overview of this field, also known as *inductive inference*, has been given by Angluin and Smith (1983).

Later, classification problems such as introduced in the formal setting of *concept learning* by Valiant (1984) became increasingly important. Given a set *X* and a set of concepts $\mathcal C \subseteq 2^{X}$, the learner has the task to identify a target concept^10^$C^*\in \mathcal C$ by evaluating training examples $x \in X$ (picked according to some probability distribution over *X*) together with a corresponding label, indicating whether *x* is contained in the target concept $C^*$. So-called PAC bounds give theoretical bounds on the number of such training examples necessary to identify the target concept *probably approximately correct*. For an overview of such results for different concept classes see Angluin (1992). For practical purposes, there is a wide range of algorithms for classification tasks available (like neural networks, decision trees, or support vector machines) some of which also come with theoretical guarantees in form of PAC bounds. Boosting approaches that combine several *weak* learners (i.e., learners that are only a bit better than random guessing) to achieve PAC learnability (Freund and Schapire 1997) are of particular theoretical interest in our context, as there are no similar techniques in reinforcement learning.

Note that both of the mentioned learning scenarios already pursue more modest aims than identifying an underlying truth in finite time. While inductive inference is interested in learning in the limit, in concept learning one is happy with an approximation of the target concept.

Comparing these settings to our reinforcement learning scenario, the latter corresponds to a *multiclass* classification^11^ task for which PAC bounds have been derived by Morvant et al. (2012)): Each history is assigned to one (unique) corresponding state. Actually, there are indeed results about reduction of performance in reinforcement learning problems to performance in (a sequence of) classification tasks (Langford and Zadrozny 2005).^12^ However, while error rates for classification tasks in this framework can be translated into regret bounds, this does not give a concrete algorithm. In particular, it is not clear how to construct training sets for the respective classifiers. For a detailed discussion of the subtleties of the reduction see (Langford and Zadrozny 2005).

Generally, compared to other machine learning settings like classification, in reinforcement learning there is the additional level of acting, with respect to which performance is measured. It is indeed a characteristic of reinforcement learning that a high probability identification (or approximation) of the underlying truth is no guarantee for high performance with respect to reward. Thus, after having identified the underlying true model after a certain number of steps with error probability $\delta >0$, it is still risky to act according to this model for the rest of the time, as with probability $\delta$ the model will be wrong and the chosen policy suboptimal. This problem is known as the “exploration vs exploitation dilemma”, which is already present in very simple reinforcement learning settings, such as the multi-armed bandit problem (Lai and Robbins 1985; Auer et al. 2002), which corresponds to a one-state MDP (with obviously trivial transition probabilities).

On the other hand, a model that achieves small regret need not be a good approximation of the underlying true model. As already indicated in Sect. 2.3, the OMS algorithm of Maillard et al. (2013) does not even try to identify or approximate the true model. Even after a large number of steps it would not be able to tell which model would give a good approximation of the true model.

### Different Notions of Model

That an algorithm can be successful without trying to identify the underlying model may be reminiscent of the discussion of Breiman (2001) about the two cultures in statistical modeling: while the traditional statistics community tends to assume an underlying (type of) model for observed data (so that only the parameters of the model have to be estimated), the machine learning community tries to solve problems algorithmically without explicitly assuming any underlying (true) model.

However, there are some notable differences to this classical setting: Most importantly, the notion of *model* Breiman (2001) uses is different from ours and rather means a *stochastic* data model that has generated the observed data. In our context, this rather corresponds to the underlying Markov decision process. The assumption that a reinforcement learning agent acts in an unknown Markov decision process is actually more in the tradition of the first culture criticized by Breiman (2001). Still, Markov decision processes are a paradigm in reinforcement learning that has seen few alternatives.^13^ Also, our assumption that the learner has access to a set of possible models containing the true model, rather corresponds to the culture that is criticized by Breiman (2001). It is still an open problem how to come up with an algorithm that either produces its models automatically or does not need any explicit models at all. As has been shown recently by Ortner et al. (2014), the assumption that the true model is part of the considered model can be relaxed if one is happy to compete with an approximate model. However, this does not imply convergence to the optimal policy (in the true model).

A related question is that of model mimicry. Thus, a different model than the true one may lead to comparably high reward even though it is not the true model (similar to Example 1). This has been discussed for statistical data models e.g. by Navarro et al. (2004) or Wagenmakers et al. (2004), where it is suggested to account for the potential of one model to imitate another one and how to measure this model mimicry. However, while Navarro et al. (2004) and Wagenmakers et al. (2004) try to identify the true data-generating model, in the reinforcement learning context this is not important as we are happy with low regret, independent of whether the model employed by the algorithm is the true one.

## Conclusion

In the considered reinforcement learning setting, it may be arguable that in cases as the one in Example 1 the question about the true model does not make sense. While we would not go as far as claiming that the concept of truth is meaningless here, in the context of reinforcement learning, truth is at least subordinate to other criteria like regret. One could interpret the situation in the sense of William James’ pragmatic theory of truth. The learner may consistently employ incorrect models which are however earning high reward. These models may be wrong from the perspective of an omniscient observer, however from the view of the learning agent they fit James’ dictum quite well:

Ideas ... become true just in so far as they help us to get into satisfactory relations with other parts of our experience (James 1907, p. 44).

## Footnotes

1 Note that the crucial property of an MDP is its *Markovian* behavior, that is, rewards and transitions only depend on the current state and not on the history.

2 In this section, *model* is not used in the sense of Definition 2 but rather refers to a model of the underlying MDP based on the estimates for rewards and transition probabilities so far.

3 That is, those parameters that maximize the optimal average reward.

4 For more discussion of the concept of “optimism in the face of uncertainty” and its limits see (Ortner 2008).

5 Such PAC bounds also exist in other learning settings, cf. the discussion in Sect. 4 below.

6 These other parameters are the size of the state and the action space as well as the *diameter*, the largest expected time it takes to reach any state from some other state. The appearance of these parameters in the bound has also been shown to be necessary. For details see (Jaksch et al. 2010).

7 While the dependence on the horizon *T* of these bounds is the same as in the MDP setting, the dependence on the other parameters is worse than in the MDP case. In particular, the bounds of Maillard et al. (2012) depend on the size of the state spaces of *all* the models. An improved bound has recently been achieved by Ortner et al. (2014). However, this new bound has worse dependence on the size of the state space and the diameter of the true model, too. Also the number of models given to the learner appears in the bound. Unlike in the MDP setting, it is still an open problem which of these parameters necessarily have to appear in any regret bound.

8 The main difference is that models in (Hallak et al. 2013) map observations (instead of histories) to states.

9 Here, a model $\phi$ is a *refinement* of a model $\phi '$ if for all observations $o_1,o_2$ it holds that $\phi (o_1)=\phi (o_2)$ implies that $\phi '(o_1)=\phi '(o_2)$. Intuitively, this means that if $\phi$ maps two observations to the same state, these observations have to correspond to the same state in the less refined model as well.

10 The target concept can be e.g. considered to be the set of instances with a certain property.

11 Unlike in *binary* classification where examples are assigned one of two possible labels (indicating the membership in the target concept), in multiclass classification there are in general more than two labels. Still, each example is assigned a unique label.

12 The idea here is however to label the histories not by the respective underlying state but by the respective optimal action.

13 Other popular settings like predictive state-representations (Littman et al. 2002) or partially observable Markov decision processes (POMDPs) (Kaelbling et al. 1998) are rather extensions of the standard MDP setting than completely independent approaches.
